# Next Generation DNA-Seq and Differential RNA-Seq Allow Re-annotation of the *Pyrococcus furiosus* DSM 3638 Genome and Provide Insights Into Archaeal Antisense Transcription

**DOI:** 10.3389/fmicb.2019.01603

**Published:** 2019-07-12

**Authors:** Felix Grünberger, Robert Reichelt, Boyke Bunk, Cathrin Spröer, Jörg Overmann, Reinhard Rachel, Dina Grohmann, Winfried Hausner

**Affiliations:** ^1^Institute of Microbiology and Archaea Center, University of Regensburg, Regensburg, Germany; ^2^Leibniz Institute DSMZ-German Collection of Microorganisms and Cell Cultures GmbH, Braunschweig, Germany; ^3^Institute of Microbiology, Technical University of Braunschweig, Braunschweig, Germany

**Keywords:** archaea, *Pyrococcus*, RNA sequencing, Nanopore sequencing, PacBio sequencing, bidirectional transcription, antisense transcription

## Abstract

*Pyrococcus furiosus* DSM 3638 is a model organism for hyperthermophilic archaea with an optimal growth temperature near 100°C. The genome was sequenced about 18 years ago. However, some publications suggest that in contrast to other *Pyrococcus* species, the genome of *P. furiosus* DSM 3638 is prone to genomic rearrangements. Therefore, we re-sequenced the genome using third generation sequencing techniques. The new *de novo* assembled genome is 1,889,914 bp in size and exhibits high sequence identity to the published sequence. However, two major deviations were detected: (1) The genome is 18,342 bp smaller than the NCBI reference genome due to a recently described deletion. (2) The region between PF0349 and PF0388 is inverted most likely due an assembly problem for the original sequence. In addition, numerous minor variations, ranging from single nucleotide exchanges, deletions or insertions were identified. The total number of insertion sequence (IS) elements is also reduced from 30 to 24 in the new sequence. Re-sequencing of a 2-year-old “lab culture” using Nanopore sequencing confirmed the overall stability of the *P. furiosus* DSM 3638 genome even under normal lab conditions without taking any special care. To improve genome annotation, the updated DNA sequence was combined with an RNA sequencing approach. Here, RNAs from eight different growth conditions were pooled to increase the number of detected transcripts. Furthermore, a differential RNA-Seq approach was employed for the identification of transcription start sites (TSSs). In total, 2515 TSSs were detected and classified into 834 primary (pTSS), 797 antisense (aTSS), 739 internal and 145 secondary TSSs. Our analysis of the upstream regions revealed a well conserved archaeal promoter structure. Interrogation of the distances between pTSSs and aTSSs revealed a significant number of antisense transcripts, which are a result of bidirectional transcription from the same TATA box. This mechanism of antisense transcript production could be further confirmed by *in vitro* transcription experiments. We assume that bidirectional transcription gives rise to non-functional antisense RNAs and that this is a widespread phenomenon in archaea due to the architecture of the TATA element and the symmetric structure of the TATA-binding protein.

## Introduction

*Pyrococcus furiosus* was isolated from geothermally heated marine sediments taken from the beach of Porto di Levante, Vulcano Island, Italy (Fiala and Stetter, 1986). It is a strictly anaerobic heterotroph, growing on maltose, starch, pyruvate, peptone and complex organic substrates. When carbohydrates are used as energy source, acetate, carbon dioxide and hydrogen are the major fermentation products (Schäfer and Schönheit, 1992; Kengen et al., 1994). In the presence of peptides, elemental sulfur is required for efficient growth and hydrogen sulfide is generated as end product. With a doubling time of only 37 min at the optimal growth temperature of 100°C, *P. furiosus* has developed to one of the best studied hyperthermophilic organisms. The first published genome sequence of *P. furiosus* DSM 3638 revealed a GC content of 40.8% and a genome size of 1.91 Mb encoding 2,225 genes and 2,122 proteins (Robb et al., 2001).

However, some publications suggest that the *P. furiosus* genome is susceptible to genomic rearrangements in comparison to the related *Pyrococcus* species *Pyrococcus abyssi* and *Pyrococcus horikoshii* (DiRuggiero et al., 2000; Brügger et al., 2002; Zivanovic et al., 2002). Genome comparison suggests that transposition events, most frequently induced by insertion sequence (IS) elements, are the major driving force for such genome variations in the *P. furiosus* genome. No full-length IS elements were identified in the genomes of the other two *Pyrococcus* species (Zivanovic et al., 2002). IS elements are short DNA sequences with a typical length between 700 and 2500 bp (Siguier et al., 2014). They contain an open reading frame (ORF) encoding a transposase, which is usually flanked by inverted repeats and promote translocation of DNA segments within and between genomes. A study analyzing the IS elements of a collection of *Pyrococcus* strains isolated from the original habitat, Vulcano Island, suggested that these elements play an important role for genetic drift in the diversification of a geographically isolated population of *P. furiosus* (Escobar-Páramo et al., 2005). Furthermore, the identification of an almost identical 16 kb region transposable region between *P. furiosus* and *Thermococcus litoralis* with only 153 nucleotide differences, indicates that these mobile elements are also involved in horizontal gene transfer (DiRuggiero et al., 2000). This region belongs to one of six highly variable chromosomal regions, which were previously identified by comparative genome hybridization using *P. furiosus* and seven *Pyrococcus* isolates from Vulcano Island (White et al., 2008). The 16 kb transposable region harbors genes encoding an ABC transport system for maltose and trehalose and is only present in *P. furiosus*, but absent from all other Vulcano isolates. This is also true for *Pyrococcus woesei*, which was isolated 1 year later from the same habitat (Zillig et al., 1987). The physiology of *P. woesei* seems to be very similar to that of *P. furiosus* and the rRNA operons of these strains have identical sequences (Kanoksilapatham et al., 2004). Although the complete genome sequence of *P. woesei* is not available, hybridization of genomic sequence from *P. woesei* to a DNA microarray of *P. furiosus* revealed the presence of additional genes in two clusters in *P. furiosus* (Hamilton-Brehm et al., 2005). One of these clusters is the 16 kb transposable region involved in the maltose metabolism. It is interesting to note that a ChIP-Seq approach from our group recently revealed the deletion of this 16 kb fragment also in *P. furiosus* and an additional southern blot analysis with a new strain from the German Collection of Microorganisms and Cell Cultures (DSMZ, Braunschweig, Germany) confirmed a rapid deletion of the fragment even with growth on maltose (Reichelt et al., 2016). Altogether, these findings support the previous suggestion to rename *P. woesei* as *P. furiosus* subsp. *woesei* (Kanoksilapatham et al., 2004).

Genome variability of *P. furiosus* strains can be observed in the natural habitat but also in strains cultivated in the laboratory. Several years of re-cultivation from stocks stored at 4°C resulted in the emergence of at least two additional *P. furiosus* strains, LS and BBR, in the Archaea Center at the University of Regensburg (Näther-Schindler et al., 2014). Both strains differ in comparison to the deposited type strain *P. furiosus* DSM 3638 in the degree of flagellation and cell morphology (Daum et al., 2017). A similar observation concerning the occurrence of a lab strain was made in Michael Adams group (University of Georgia, Athens, Georgia). This lab strain exhibits an extended exponential growth phase and atypical cell aggregation behavior (Lewis et al., 2015). The genome sequence of the mutant showed 145 genes with one or more insertions, deletions or substitutions. The data clearly demonstrate that *P. furiosus* has most likely due to the presence of IS elements an inherent property for efficient genome rearrangements. This facilitates the selection of special mutants under a proper selection pressure, but also stimulates the non-specific accumulation of different mutations within the genome. The best example for this process is the development of the genetically tractable *P. furiosus* strain COM1 (Bridger et al., 2012). The genome sequence of this strain is 1,571 bp longer than the type strain and contains numerous chromosomal rearrangements, deletions, and single base changes, which lead to the inactivation of 20 genes and to amino acid sequence variations of another 102 gene products. These changes impact various cellular functions including a riboflavin requirement for growth. The alignment of the COM1 genome sequence with the published *P. furiosus* genome revealed major inversions, but an additional analysis of the chromosomal orientations of the original DSMZ strain (ordered in October 2010) by PCR showed that some of this major inversions are also present in the original DSMZ strain (Bridger et al., 2012).

The *P. furiosus* genome sequence was published 18 years ago, but sequencing technologies and bioinformatic analysis have been revolutionized during the last 10 years. The introduction of massively parallel sequencing led to a significant reduction of sequencing costs. However, these so-called second-generation sequencing techniques produce only short reads, which impedes the assembly of the complete genome as repetitive regions cannot be resolved (Verma et al., 2017). Meanwhile, third-generation sequencing techniques have also entered the market. These systems act directly on the native DNA without the requirement for PCR amplification and show a significant increase in read length, which facilitates complete genome assemblies.

The discrepancy between the published *P. furiosus* genome sequence (Robb et al., 2001) and the detected deviations in the genome from a recently ordered *P. furiosus* strain DSM 3638 from the DSMZ (Bridger et al., 2012; Reichelt et al., 2016) and the fact that many groups make use of the originally described *P. furiosus* strain, encouraged us to re-sequence the type strain *P. furiosus*. To address the problem concerning the observed genome rearrangements and to allow for complete genome assembly we used a hybrid approach of third generation long-read PacBio sequencing complemented with highly accurate short-read Illumina sequencing (Rhoads and Au, 2015). We amended the DNA-sequencing approach with differential RNA sequencing data, to generate a high-resolution annotation using the ANNOgesic pipeline (Sharma et al., 2010; Yu et al., 2018). Last but not least, to gain insight into the genomic variability of continuously cultivated lab strains and to investigate, if it is possible to maintain genome stability by avoiding strong selection pressure during cultivation, we re-sequenced *P. furiosus* again after 2 years of cultivation employing the recently developed Nanopore sequencing technology (Loman et al., 2015). Our results indicate a quite stable genome even with the strain cultivated for 2 years in the lab and differential RNA-Seq data revealed that bidirectional transcription is a significant source for archaeal antisense transcripts.

## Materials and Methods

### Strains and Growth Conditions

*Pyrococcus furiosus* strain DSM 3638 was stored as freeze-dried culture at 12°C in the dark at the *Deutsche Sammlung von Mikroorganismen und Zellkulturen* (DSMZ) in Braunschweig, Germany. For the isolation of DNA for combined PacBio and Illumina sequencing, cells were grown anaerobically in 20 ml SME medium supplemented with 0.1% yeast extract and 0.1% starch at 95°C to late-exponential phase (Fiala and Stetter, 1986).

For Nanopore sequencing, a culture was obtained from the DSMZ in 2015 and after growth in SME media the strain was stored in the gas phase of liquid nitrogen at the archaea center in Regensburg for 1 year. After that, the strain was recultivated and handled in the lab for about 2 years with numerous inoculations, to simulate storage and daily life usage conditions of many labs with a focus on microbiology. We assume that during these 2 years the culture was about -roughly estimated- thirty times transferred into fresh medium. In between, liquid cultures were stored at room temperature or 4°C. For the isolation of DNA cells were grown anaerobically in 40 ml SME medium supplemented with 40 mM pyruvate, 0.1% peptone and 0.1% yeast extract at 85°C to mid-exponential phase.

For RNA sequencing *P. furiosus* cells were grown under eight different conditions to maximize the number of different transcripts in the genome: Cells were grown anaerobically in 20 ml SME medium supplemented with 0.1% starch, 0.1% peptone and 0.1% yeast extract at 95°C to early- (1 × 10^7^ cells/ml), mid-exponential (5 × 10^7^ cells/ml) or late-exponential (1 × 10^8^ cells/ml) phase (conditions 1, 2, and 3). In addition, cells were grown in 20 ml SME medium supplemented with 0.1% starch, 0.1% peptone and 0.1% yeast extract at 95°C to late-exponential phase, further incubated at 4°C for 1 h (condition 4; cold shock) or 110°C for 15 min (condition 5; heat shock). Moreover, cells were grown anaerobically in 20 ml SME medium supplemented with 0.1% starch, 0.1% peptone and 0.1% yeast extract at 75°C to late-exponential phase (condition 6; cold adaption). Furthermore, cells were grown anaerobically in 20 ml SME medium supplemented with 0.1% yeast extract and 0.1% starch (condition 7; glycolytic growth) or 40 mM pyruvate (condition 8; gluconeogenic growth) at 95°C to late-exponential phase. Cells were harvested by centrifugation at 3,939 × *g* for 45 min at 4°C, cell pellets were frozen in liquid nitrogen and stored at −80°C until used for the isolation of DNA or RNA.

### DNA Isolation

Genomic DNA was isolated using ReliaPrep^TM^ gDNA Tissue Miniprep System (Promega) according to the instructions of the manufacturer. Quantity and quality were analyzed using Nanodrop One, Qubit dsDNA HS assay kit (both from Thermo Fisher Scientific) and agarose gel electrophoresis. For Nanopore sequencing size distribution was checked using pulsed field gel electrophoresis on a CHEF-DR^®^III system (Bio-rad).

### RNA Extraction

*P. furiosus* total RNA was purified using the peqGOLD TriFast^TM^ reagent (VWR). 20 ml cell culture was pelleted, and cells were lysed by addition of 1 ml TriFast followed by rigorous shaking for 15 min. After adding 0.2 ml 2 M sodium acetate pH 4.0 RNA was isolated according to the manufacturer instructions. Contaminating DNA was removed via the TURBO DNA-free^TM^ Kit (Thermo Fisher Scientific, Waltham, MA, United States). The integrity of the total RNA was assessed via a Bioanalyzer (Agilent) run using the RNA 6000 Pico Kit (Agilent) and purified RNA was stored at −80.

### PacBio Library Preparation and Sequencing

SMRTbell^TM^ template library was prepared according to the instructions from Pacific Biosciences, Menlo Park, CA, United States, following the Procedure and Checklist – Greater Than 10 kb Template Preparation. Briefly, for preparation of 15 kb libraries 8 μg genomic DNA was sheared using g-tubes^TM^ from Covaris, Woburn, MA, United States according to the manufacturer’s instructions. DNA was end-repaired and ligated overnight to hairpin adapters applying components from the DNA/Polymerase Binding Kit P6 from Pacific Biosciences, Menlo Park, CA, United States. Reactions were carried out according to the manufacturer’s instructions. BluePippin^TM^ Size-Selection to greater than 7 kb was performed according to the manufacturer’s instructions (Sage Science, Beverly, MA, United States). Conditions for annealing of sequencing primers and binding of polymerase to purified SMRTbell^TM^ template were assessed with the Calculator in RS Remote, Pacific Biosciences, Menlo Park, CA, United States. SMRT sequencing was carried out on the PacBio *RSII* (Pacific Biosciences, Menlo Park, CA, United States) taking one 240-min movie on two SMRT Cells.

### Genome Assembly, Error Correction, and Annotation

SMRT Cell data was assembled using the “RS_HGAP_Assembly.3” protocol included in SMRT Portal version 2.3.0 using default parameters. The assembly revealed a single circular chromosome. The chromosome was circularized, particularly artificial redundancies at the ends of the assembled contig were removed and adjusted to *cdc6* as the first gene. Error-correction was performed by a mapping of paired-end reads of 2 × 100 bp generated on an Illumina HiSeq 2500 onto finished genomes using BWA (Li and Durbin, 2010) with subsequent variant and consensus calling using VarScan (Koboldt et al., 2012). A consensus concordance of QV60 could be confirmed. Finally, an annotation was carried out using NCBI prokaryotic genome annotation pipeline (Tatusova et al., 2016). The genome sequence was deposited in NCBI GenBank under Accession Number CP023154.

### Nanopore Sequencing (MinION)

#### Library Preparation and Sequencing

Library preparation was performed according to Oxford Nanopore Technologies (ONT, Oxford, United Kingdom) protocol for multiplexing samples (1D native barcoding genomic DNA with EXP-NBD103 and SQK-LSK108). Sequencing was performed on ONT’s MinION MK1B device, connected to a laptop computer via USB3, using MinKNOW software (v.1.4.3). After confirmation of a sufficient number of active pores on the flow cell (SpotON Flow cell Mk I R9 version), the prepared DNA library was loaded onto the flow cell and sequencing was started choosing the 48 h sequencing protocol suggested by MinKNOW.

#### Basecalling, *de novo* Assembly, Polishing and Evaluation

For MinION data analysis raw reads in fast5 data format were base-called and de-multiplexed using Albacore 2.3.4. In a first step a *de novo* genome assembly was done using canu 1.8 (genomeSize = 1.9 m, minReadLength = 500, minOverlapLength = 100) (Koren et al., 2016), before improving the consensus sequence in a second step with *nanopolish* 0.11 (min-candidate-frequency = 0.1) (Simpson et al., 2017). The chromosome was circularized, artificial redundancies at the ends of the assembled contig removed and adjusted to *cdc6* as the first gene (compare 2.5). To determine the identity of the *de novo* assembly to the hybrid PacBio-Illumina approach, statistics from dnadiff (MUMmer version 3) were calculated and visualized using R package *genoPlotR* (Kurtz et al., 2004; Guy et al., 2011). Read length and nucleotide frequencies were analyzed using the statistical program R with ggplot2 (R Development Core Team, 2011; Wickham, 2016). Code is available at: https://github.com/felixgrunberger/pyrococcus_reannotation.

### Illumina Sequencing (RNA-Seq)

RNAs purified from cells grown under eight different growth conditions were pooled equally and submitted for library preparation and sequencing to the Core Unit Systems Medicine (SysMed) at the University of Würzburg, Germany. Three different libraries were prepared to fulfill the requirements for usage in the ANNOgesic pipeline: fragmented, unfragmented with terminator exonuclease treatment (+TEX) and unfragmented without TEX-treatment (−TEX). For the fragmented sample, RNA was fragmented for 2 min at 94°C using the NEBNext^®^Magnesium RNA Fragmentation Module. Afterward RNA was treated with T4 Polynucleotide Kinase (PNK) without ATP for 6 h at 37°C and 1 h at 37°C with 2 mM ATP and fresh T4 PNK. After overnight ethanol precipitation, 5′ triphosphates were removed using RNA 5′ Pyrophosphohydrolase (RppH) for 1 h at 37°C. RNA was again precipitated and resuspended in 6 μl H_2_O. The two samples (+/− TEX) for the transcription start site detection were either treated with TEX (+ TEX) or with H_2_O as a mock control (− TEX) for 30 min at 37°C. Afterward both samples were treated with RppH for 1 h at 37°C before they were precipitated, and the RNA was resuspended in 6 μl H_2_O. After the pre-treatment, all three libraries were prepared using the NEBNext^®^Multiplex Small RNA Library Prep Kit for Illumina according to the manufacturer’s protocol with small modifications. The first linker ligation was performed for 18 h at 16°C and libraries were amplified with 12 PCR cycles with an extended elongation time of 75 s. Libraries were pooled in a 2:1:1 ratio (fragmented: + TEX : − TEX) and sequenced on an Illumina NextSeq 500 high-output single-end 75 nt run.

### Trimming and Mapping of RNA-Seq Reads

Illumina reads in FASTQ format were quality/length/adapter trimmed using *trimmomatic* (v.0.36) in single-end-mode allowing for a minimum length of 12 nt and a cut-off Phred score of 20, calculated in a sliding window of 4 bases (Bolger et al., 2014). Next, reads were mapped using STAR aligner (v.2.5.3) to the new assembled genome of *P. furiosus* (Dobin et al., 2013). Mapping statistics (input, filtered, uniquely aligned reads) can be found in the Supplementary Table 3. To use ANNOgesic for RNA-based annotation of *P. furiosus*, strand-specific coverage files in wiggle format of all three sequencing data sets were generated (Yu et al., 2018). As recommended, reads were additionally mapped with segemehl 0.2.0 to detect circular RNAs within the ANNOgesic pipeline (Otto et al., 2014).

### Reference Genome Annotation Using ANNOgesic

ANNOgesic is a recently published pipeline that predicts transcriptome-wide features based on a combination of differential and fragmented RNA sequencing (Yu et al., 2018). Amongst others, it is built on TSSpredator, using adaptive parameter optimization, which simplifies and improves detection of transcription start sites (TSS) (Dugar et al., 2013). Following subcommands were executed in the provided Docker image of ANNOgesic to improve annotation of *P. furiosus* (basic parameters if not stated otherwise): optimize_tss_ps (with 50 manually detected TSS as a reference, 4000 iterations), tss_ps (with optimized parameters from previous step), transcript, terminator, utr, operon, srna, sorf, circrna (cut-off supported reads: 200), promoter, crispr. Features in gff file format were combined with the merge_features command and added to the gff file from DNA sequencing and assembly (Supplementary Table 5).

### RNA-Seq Data Analysis

Data analysis of output files from ANNOgesic was done using the R/Bioconductor environment (R Development Core Team, 2011). Scripts for analysis were uploaded to https://github.com/felixgrunberger/pyrococcus_reannotation.

#### Detection of Promoter Elements

For the detection of common archaeal promoter elements, a position weight matrix (PWM) was calculated from sequences 50 bases upstream to 10 bases downstream of all available TSS. The resulting motif was visualized in R using ggseqlogo (Wagih, 2017).

The sequences 51 bases upstream of every TSS were extracted to identify the best ranking promoter motif for each TSS category using MEME with default options except “search given strand only” (Bailey et al., 2009). Motifs and position tables were further analyzed using ggseqlogo and ggplot2. For internal TSS a repetitive sequence coming from CRISPR regions gave the best motif but was excluded from further analysis.

Length of 5′ UTRs of pTSS and sTSS was already calculated in the ANNOgesic pipeline and visualized using ggplot2. Internal and antisense TSS positions relative to a gene were sorted in three windows: 300 bp upstream, 300 bp downstream and in between annotated genes. Positions between start and stop site were scaled according to gene length.

To find a motif for possible bidirectional promoters we filtered all primary TSS that had strong TEX signal on the antisense strand (more than 40% of the reads from −400 to +400 in the region 100 bp upstream of pTSS) and calculated a motif using MEME (default options).

#### Coverage Plots

We generated average coverage profile plots to check for the enrichment of TSS in the TEX data set and to validate the TSSpredator classification. The R package CoverageView was used to calculate the coverage for each sequencing data set from 400 bp upstream and downstream relative to a TSS in a window of 10 bp (Lowy, 2017). Every position for a single TSS was scaled proportionally, before calculating mean values for plotting.

Coverage plots were also generated for the analysis of putative bidirectional promoters. A similar protocol as mentioned above was used. We split the data set into two groups (head-to-head and head-to-tail) considering the orientation of the upstream gene. From this data set we also calculated the intergene distance for annotated genes.

#### Antisense Enrichment Around IS Elements

Insertion sequence elements for the available *Pyrococcus* assemblies were identified using ISEScan 1.6 (Xie and Tang, 2017). Genomic positions of these elements were extracted to scan for antisense TSS nearby. The relative position was calculated in a window 100 bp upstream from the start of an IS element, 100 bp downstream from the end and in between scaled according to length. The aTSS and IS elements used for the analysis to create Figure 8B are listed in Supplementary Table 6.

### *In vitro* Analysis of Bidirectional Transcription

For the analysis of bidirectional transcription *in vitro* transcription reactions were assembled in 100 μl reaction volumes (40 mM Na-HEPES, pH 7.3, 0.1 mM EDTA, 0.1 mg/ml BSA, 2.5 mM MgCl_2_, 250 mM KCl): 8.8 nM DNA was combined with 190 nM TBP, 105 nM TFB, 108 nM TFE, and 10.5 nM RNA polymerase. The 349 bp DNA fragment was prepared by PCR with the primers 5′-gaaagggcgaaccagttagattgaacg and 5′-tgttgggcttctccccaagctgag using genomic DNA as template. Transcription was initiated by the addition of NTPs to a final concentration of 100 μM and reactions were incubated at 80°C for 10 min. Reactions were stopped by extraction with one volume phenol/chloroform/isoamyl alcohol, RNA was precipitated with ethanol and resuspended in 20 μl H_2_O. For the analysis of the transcripts primer extension experiments were carried out using labeled primers in sense or antisense direction. 10 μl *in vitro* RNA were combined with 125 nM of the corresponding primer in a total volume of 15 μl. After RNA denaturing at 70°C for 5 min, primer annealing was performed at 0°C for 5 min. Primer extension was started by the addition of 5 μl reverse transcription mixture containing 50 units of M-MLV RNase H minus (Promega) and 1 mM dNTPs. After incubation at 48°C for 30 min, cDNA products were purified by ethanol precipitation, resuspended in 10 μl formamide buffer and analyzed on a 20% denaturing polyacrylamide gel. The DNA fragments were visualized with a ChemiDoc MP imaging system (Bio-rad).

## Results and Discussion

### Strategy for Genome Re-annotation of *Pyrococcus furiosus*

**FIGURE 1:**
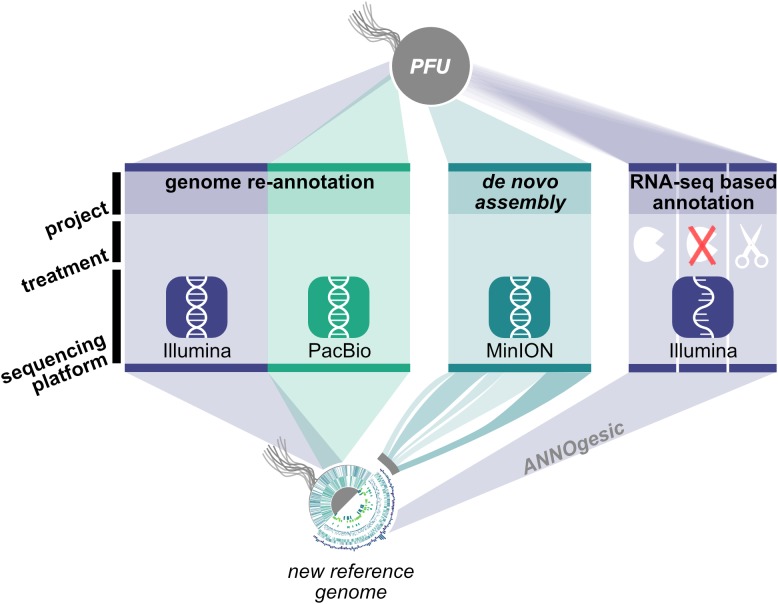
Workflow for genome re-annotation of *P. furiosus*. To build the new reference genome of *P. furiosus* DSM 3638 we used a hybrid PacBio-Illumina approach. After 2 years of subcultivation genome stability of the same strain was tested using Nanopore MinION sequencing and *de novo* assembly. Genome annotation was improved with an RNA-Seq based approach of eight mixed growth conditions to cover a broad range of transcripts. Three different RNA treatments (terminator-exonuclease treated, not-treated, fragmented) were used to map transcription start sites and additional features using the ANNOgesic pipeline (Yu et al., 2018).

In order to address the described questions concerning the stability of the *Pyrococcus* genome, we employed a combination of DNA and RNA sequencing techniques to generate an updated version of the *P. furiosus* genome (Figure 1). We used the current gold standard in genome assembly approaches, a combination of long read PacBio sequencing and short read Illumina sequencing, to obtain a highly accurate reference genome of QV60 (<1 error per Mbp) for further analysis. Differential RNA sequencing was used to map primary transcription start sites (TSS) and to improve genome annotation with the recently developed ANNOgesic pipeline (Yu et al., 2018). In order to test whether *P. furiosus*’ genome is subject to genome instability upon long-term cultivation of the strain, we re-sequenced a 2-year-old lab culture of *P. furiosus* employing the Nanopore sequencing technique.

### Genome Comparison

#### A New Reference Genome With Two Major Deviations

**FIGURE 2:**
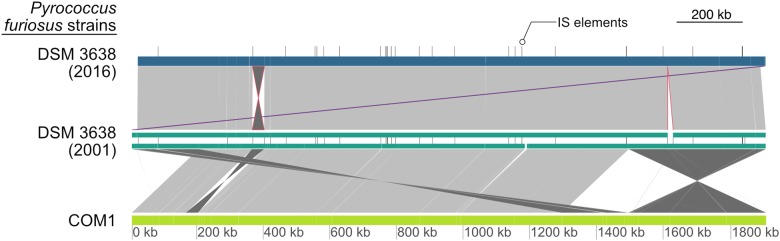
Global pairwise comparison of the genome organization of the new and the original *P. furiosus* DNA sequence together with the COM1 strain (Bridger et al., 2012). Whole genome alignments were calculated with dnadiff and visualized using GenoPlotR (Kurtz et al., 2004; Guy et al., 2011). Each genome is shown as colored blocks (blue, green, light green), whereas IS elements of DSM 3638 are indicated by vertical lines. Direct matches with high similarity between genomes are colored in light gray and inversions in dark gray. The inversion of the fragment from PFDSM3638_01715 to PFDSM3638_01910 and the deletion of the 17,075 bp fragment are indicated in red and the adjusted annotation start to *cdc6* as the first gene is highlighted in purple. Additional minor variations are below the resolution of the presented map. In contrast to the large genomic rearrangements between the COM1 strain and the type strain of *P. furiosus*, there are only minor differences between the old and the new DNA sequence of *P. furiosus* DSM 3638.

Based on PacBio sequencing data that provided a 194-fold coverage of the genome, the *P. furiosus* type strain DSM 3638 genome was assembled *de novo* to a single contig sequence, which was error-corrected by Illumina data. The comparison with the current NCBI reference sequence (NC_003413) revealed that the new genome sequence (CP023154) is strongly syntenous to the published sequence (Figure 2, upper part). However, we found two major variations:

(1) The fragment encoding the genes PFDSM3638_01715 to PFDSM3638_01910 is inverted in comparison to the corresponding region PF_RS01790 (PF0349) to PF_RS01990 (PF0388) in the original reference sequence NC_003413. This inversion represents one of numerous described chromosomal rearrangements identified for the *P. furiosus* strain COM1 (Figure 2, lower part) (Bridger et al., 2012). But in contrast to the other differences this deviation was also found in the sequencing data of a newly ordered type strain from the DSMZ by the same group (Bridger et al., 2012). This indicates that this inversion is most likely caused by an assembly problem of the original sequence (Robb et al., 2001).
(2) The region from 1,613,139 to 1,630,214 is deleted in the re-sequenced genome. The deletion of this region has also been identified previously by a ChIP-Seq approach (Reichelt et al., 2016). It belongs to a highly variable chromosomal region, which is flanked by two IS elements and proposed as an example for a recent transposon mediated gene transfer between *P. furiosus* and *Thermococcus litoralis* (DiRuggiero et al., 2000). The fragment encodes a trehalose/maltose-specific-ABC-transporter. Even growth on maltose could not prevent this deletion (Reichelt et al., 2016).

Due to the above-mentioned deletion and a reduced number of IS elements, the complete sequence consists of 1,889,914 bp, which is 18,342 bp smaller than the NCBI reference genome (Figure 1, upper part, and Table 1). The GC content and the total number of genes are also slightly reduced due to the deletion events. Using the NCBI prokaryotic genome annotation pipeline (Tatusova et al., 2016), the new genome sequence harbors 2,035 genes of which 1,982 encode proteins, whereas the residual 68 genes transcribe four rRNA genes (one 23S rRNA, one 16S rRNA and two 5S rRNAs), 46 tRNAs and 18 additional ncRNAs. The values are very similar in comparison to the annotations from the other two assemblies (Table 1). Moreover, the number of pseudogenes decreased in the new assembly from 74 to 53, which indicates that the re-sequencing allowed for correction of frameshifted genes now being present correctly annotated in full-length. All three genomes harbor seven CRISPR arrays, but the number of spacers in the CRISPR array 6 differs between the new and old *P. furiosus* type strain DSM 3638 assemblies (36 vs. 21). This might be due to assembly problems of repetitive sequences in the initial sequencing in 2002. In addition, numerous minor variations were identified including single nucleotide exchanges (causing silent or missense mutations), frameshift insertions or deletions and deletions or insertions of complete genes (summarized in Supplementary Table 1). Some of these variations were already reported by previous studies. For example, the *flaB0* gene was discovered in an earlier study, which encodes the major flagellin of the *P. furiosus* archaellum apparatus (Näther-Schindler et al., 2014). A comparison of all coding sequences from the annotation based on the new genome assembly (CP023154) with the annotation based on the genome assembly (NC_003413) from 2002 is shown in the Supplementary Table 2.

**Table 1:** Genome comparison of the re-sequenced Pyrococcus furiosus DSM 3638 together with the first published NCBI reference sequence (NC_003413) and P. furiosus COM1 (NC_018092).

NCBI GenBank Accession	DSM 3638_2016 CP023154	DSM 3638_2001 NC_003413	COM_1 NC_018092
Genome length (bp)^1^	1889914	1908256	1909827
GC content [%]	40.75	40.77	40.79
Genes (total)^1^	2,035	2,053	2,066
Genes (coding)^1^	1,982	1,979	2,001
Genes (RNA)^1^	68	68	67
complete rRNAs^1^	2, 1, 1	2, 1, 1	2, 1, 1
	(5S, 16S, 23S)	(5S, 16S, 23S)	(5S, 16S, 23S)
tRNAs^1^	46	46	46
ncRNAs^1^	18	18	17
Pseudo Genes (total)^1^	53	74	65
CRISPR Arrays^1,2^	7	7	7
CRISPR1 Spacer^2^	51	51	51
CRISPR2 Spacer^2^	21	20	21
CRISPR3 Spacer^2^	23	22	23
CRISPR4 Spacer^2^	30	30	30
CRISPR5 Spacer^2^	45	45	44
CRISPR6 Spacer^2^	36	21	36
CRISPR7 Spacer^2^	11	11	11
Total no. of IS elements^3^	24	30	40
IS200/IS605^3^	1	1	1
IS6^3^	17	23	33
IS982^3^	5	5	5
new^3^	1	1	1

*^1^NCBI Prokaryotic Genome Annotation Pipeline (Tatusova et al., 2016). ^2^CRISPRFinder (Grissa et al., 2007). ^3^ISEScan (Xie and Tang, 2017).*

#### Nanopore Sequencing Confirms Genome Stability

Re-sequencing of the type strain, which was stored for more than 15 years under optimal conditions at the DSMZ, indicated indeed a very stable genome over the years. But is this also true for a strain handled in the lab which is repeatedly inoculated and stored over time in liquid cultures? To answer this question, we re-sequenced the “lab culture” about 2 years after we performed the Illumina/PacBio sequencing employing this time the recently developed Nanopore sequencing technique. During these 2 years the culture was about -roughly estimated- thirty times transferred into fresh medium. A total of 397,582 reads were accumulated of which 328,862 (82.7%) had a mean Phred-based quality score (qscore albacore) equal or better than 7.0 representing ∼308-fold genome coverage. The median Phred quality score for all reads used for further assembly steps was 8.82.

We performed a *de novo* assembly of the genome based on the Nanopore sequencing data. First, we generated a draft genome using Canu. Subsequently, we improved the consensus sequence with Nanopolish (Koren et al., 2016; Simpson et al., 2017). In the first step, we were able to reach a closed assembly with 1 contig (1,891,829 bp) with no genomic rearrangements observed compared to the PacBio/Illumina reference sequence. In general, the genome was only 0.1% assembled larger with an identity of 99.42% compared to the reference genome (Table 2). After polishing, the sequence identity further improved to 99.92%. Most of the additional base pairs (+0.35%) can be explained by insertions throughout the genome with a slight preference for additional As and Ts (A: 27%, T: 27% of all insertions).

**Table 2:** Nanopore sequencing is suitable for generating a high identity genome *de novo* in comparison with hybrid Illumina/PacBio data.

Assembly	Total Bases	No. of contigs	GC content	% Identity (1-to-1 dnadiff mummer)
Illumina/PacBio	1889914	1	40.75	100
Nanopore raw (Canu)	1891829	1	40.69	99.42
Nanopore polished (Nanopolish)	1896610	1	40.75	99.92

*A closed genome (No. of contigs = 1) from Oxford Nanopore data was reached with Canu. One round of Nanopolish was used to further increase sequence identity.*

**FIGURE 3:**
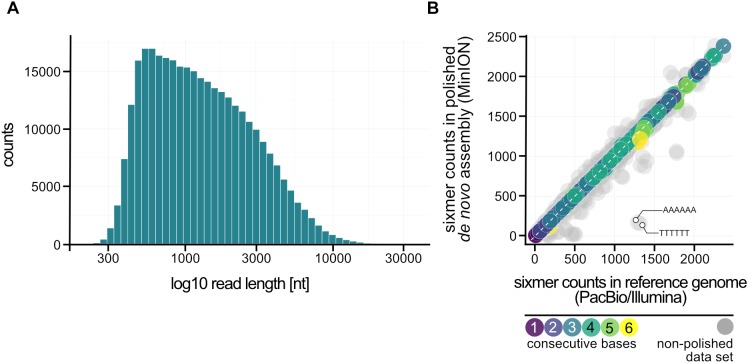
Nanopore sequencing of *P. furiosus*. **(A)** Fragmentation of the DNA due to the DNA purification protocol used can be ascertained from the read length distribution after sequencing (median: 1,160 bp). **(B)** Sixmer-comparison of PacBio/Illumina assembly to Nanopore *de novo* assembly (polished: colored-, not-polished: gray) shows known drawbacks of Nanopore-sequencing with limited resolution of homopolymers that requires bioinformatic polishing (Simpson et al., 2017).

One major advantage of the Nanopore technique is the significantly increased read length in comparison to Illumina sequencing, which facilitates genome assembly and helps to identify genome rearrangements. The usage of a column-based DNA purification protocol led to a fragmentation of DNA, which can be observed in the length distribution of sequenced reads (median length: 1,160 bp; longest read: 31,965 bp) (Figure 3A). Most of the errors in the non-polished assembly were in fact not random but can be explained when the counts of sixmers (all combinations of 6 nucleotides) in both assemblies are analyzed (Figure 3B). Bioinformatical polishing successfully reduced the differences in sixmer usage and lead to a very high sequence identity. All in all, we could not observe any large differences in genome organization after 2 years on/off cultivation and storage. Therefore, we conclude that the *Pyrococcus* genome is more stable than previously expected.

### Analysis of the Primary Transcriptome

We combined the updated DNA sequence with an RNA sequencing approach to improve genome annotation with the recently developed ANNOgesic pipeline (Yu et al., 2018). To maximize the number of detected transcripts in the *P. furiosus* genome we pooled RNA preparations from eight different growth conditions. In one dataset we fragmented the RNA before generation of cDNA libraries to increase RNA coverage. For the enrichment of primary transcription start sites (TSS), we employed the differential RNA-Seq (dRNA-Seq) approach, which uses a terminator exonuclease (TEX) treatment to degrade RNAs that exhibit a 5′ monophosphate that arise from nucleolytic degradation of primary transcripts but not RNAs with a 5′ triphosphate (Sharma et al., 2010). A TEX-untreated cDNA library served as a control, which includes in addition the 5′ ends of processed or degraded RNAs. Sequencing and mapping statistics can be found in Supplementary Table 3.

**FIGURE 4:**
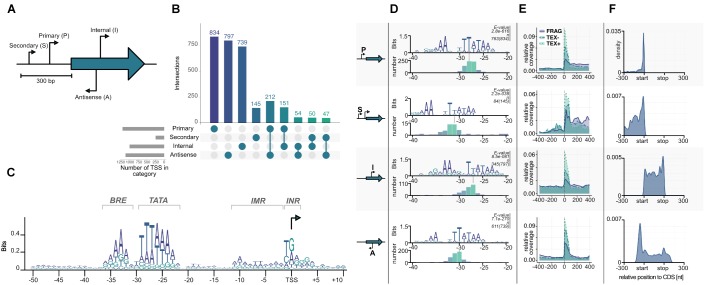
Transcription start site (TSS) classification. **(A)** Primary (P), Secondary (S), Internal (I), and Antisense (A) TSSs are classified by TSSpredator according to their position relative to the next gene (Dugar et al., 2013). **(B)** Number of TSS identified in each category after using adaptive parameter optimization from ANNOgesic (Yu et al., 2018). **(C)** Known archaeal promoter elements can be detected by visualizing a PWM calculated from all TSS from −50 to +10 bases to a start site. **(D)** Promoter motifs for the individual TSS categories identified by MEME search of all upstream sequences (−50, +1). The *e*-values and the number of sequences contributing to each motif are shown on top of each panel. The panels at the bottom of each category shows a histogram aligned to the third T of the corresponding TATA boxes. **(E)** Relative coverage of all reads in three sequencing datasets fragmented (FRAG, purple), terminator-exonuclease treated (TEX, blue) and TEX-non-treated-control (NOTEX, green) are plotted in relative position to a TSS in a window of −400 to +400 to confirm the output of the classification algorithm. **(F)** Position of all TSS according to next gene with normalized length in a window of −300 to annotation-start and +300 to annotation-end (on gene level) are plotted. Primary TSS have predominantly short 5′ UTR lengths with a median of 13 nt, secondary TSS have larger 5′ UTRs and internal TSS are equally distributed across the corresponding gene length. Antisense TSS seem to be enriched in gene-flanking regions.

Transcription start sites were identified within the ANNOgesic pipeline using TSSpredator and classified into 834 primary (P), 797 antisense (A), 739 internal (I), and 145 secondary (S) transcripts according to their position relative to the next gene (Figure 4A,B; Dugar et al., 2013; Yu et al., 2018). After using an iterative optimization process in the parameter selection module of the newly developed TSSpredator (Yu et al., 2018), the total number of TSS is similar to previously published archaeal primary transcriptome sets considering different genome sizes (Jäger et al., 2009, 2014; Babski et al., 2016; Cho et al., 2017; Smollett et al., 2017). As a result of the densely packed genome, some of these identified transcripts belong to more than one category. For example, 212 TSS were categorized both as pTSS and aTSS that arise from head-to-head oriented genes.

Using Operon-mapper we analyzed in more detail how transcription of the 2035 identified genes is organized (Taboada et al., 2018). The program recognized 953 transcription units, which consist of 501 single genes and 452 operon structures, which contain the residual 1534 genes. One half (760) is organized in operons with two or three genes and the other half (774) is located in more complex operons with four or more genes (Supplementary Table 4). A comparison of the pTSS with the 953 transcription units revealed that almost 70% (571) of the identified primary transcripts match perfectly with the identified transcription units. It has to be emphasized that the *in silico* based operon prediction is purely based on intergenic distances and functional relationships between the genes (Taboada et al., 2018). Using a multiple conditions approach, we were able to use high-resolution transcriptomic data to improve current annotation by predicting operons this time based on TSSs, transcripts and genes within the ANNOgesic pipeline (Yu et al., 2018). The total number of transcription units decreased to 693 with the great majority (473, 68.25%) being identical to the Operon-mapper predicted units. The transcriptomic-based detection of transcription units reflects the true biological organization rather than an artifact from annotation, because we were able to detect every single gene in the fragmented RNA-Seq dataset (minimum number of reads per gene: 69).

We also analyzed in more detail the promoter structure of these transcription units. In general, most archaeal promoters consist of three conserved parts, TFB recognition element (BRE), TATA box and a pyrimidine/purine dinucleotide (INR) at the −1/+1 position of the TSS (Hausner et al., 1991; Soppa, 1999; Van De Werken et al., 2006). A position weight matrix (PWM) calculated in a region −50 to +10 from all TSS confirmed the presence of a highly conserved promoter structure with consensus sequences for the BRE −36(RRAAA)−32, the TATA box −30(WTTTAAAW)−23, and the INR −1(YR)+1 (Figure 4C). It is also possible to identify the initially melted region from −11 to −2 which facilitates open complex formation due to accumulation of AT sequences. The identified promoter for *P. furiosus* fits well with published data of related organisms, e.g., *P. abyssi*, *Thermococcus kodakarensis* or *Thermococcus onnurineus* NA1 (Toffano-Nioche et al., 2013; Jäger et al., 2014; Cho et al., 2017). This is also an additional indication that the TEX treatment was successful and all the identified TSS indeed represent initiation start points of the RNA polymerase. To answer the question if different promoter structures are used in the case of secondary, internal or antisense transcripts, the sequences from −50 to +1 of all identified TSS were also individually analyzed using *MEME* for identification of the best fitting motif (Figure 4D; Bailey et al., 2009). All motifs exhibit typical BRE and TATA box sequences, but with reduced conservation for secondary, internal and antisense TSS. Furthermore, the location of the TATA box in relation to the TSS is slightly different. In the case of pTSS the last conserved adenine nucleotide of the TATA box is 23 bp upstream of the TSS, which is in perfect agreement with the consensus sequence of all TSS (Figure 4C) and with published data (Toffano-Nioche et al., 2013; Jäger et al., 2014;Cho et al., 2017). In contrast, the position for sTSS is at −27, for iTSS at −22 and for aTSS at −25. From previous *in vitro* transcription experiments it is known, that these distances still enable transcription, but most likely with a reduced efficiency at least for the sTSS and aTSS (Hausner et al., 1991). To further validate the identification of different TSS classes, we analyzed the distribution of reads in a window from −400 to +400 bp of all annotated TSSs (Figure 4E). As expected, we observed the highest enrichment for the TEX dataset in all TSS classes, confirming a successful enzymatical treatment and downstream bioinformatical analysis. To exclude any further bias and gain insights into possible different regulation mechanisms of the four TSS groups, we plotted the positions of all TSS 300 bp upstream and downstream of the corresponding coding sequences (Figure 4F). Most of the transcripts initiate in close proximity to the coding sequence (pTSS, median: 13 nt, mean: 49.17 nt), indicated by the strong peak of the pTSS near the start codon in contrast to sTSS that exhibit significantly longer 5′ UTR sequences. iTSS are equally distributed over the whole gene length with one prominent peak at the end of the coding sequence. Due to the high gene density in *P. furiosus* it is possible that some of these iTSS represent pTSS of downstream genes. This is also true for the strong peak upstream of the coding sequence within the aTSS. In general, the distribution of 5′ untranslated regions (5′ UTR) is similar to the data published in the *Thermococcales* (Jäger et al., 2014; Cho et al., 2017), but quite different from predominantly leaderless transcripts in *Haloferax volcanii* (Babski et al., 2016) and long untranslated leader regions in *Methanocaldococcus jannaschii* (Smollett et al., 2017; Supplementary Figure 1).

### Characterization of Bidirectional Transcription in the Context of aTSS

**FIGURE 5:**
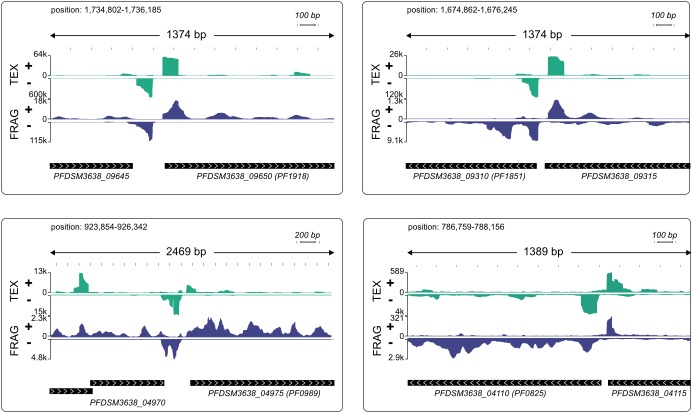
Modified IGV snapshots from head-to-tail genes with an antisense TSS in close proximity to a pTSS. The exact positions of each fragment within the genome are indicated. The genes are annotated with the new genome locus tags and with old locus tags in brackets. RNA coverage on both strands is autoscaled to fit the window, with sequencing depth indicated on the *y*-axis, TEX-treated RNA in green and fragmented RNA in blue.

It is interesting to note the high number of aTSS which is in agreement with data from other archaeal organisms (Babski et al., 2016). The function of these transcripts is not known, but we assume that at least some of them are most likely nonsense transcripts, which arise from symmetric promoter sequences. A closer look to the consensus sequence (Figure 4C) revealed an almost symmetric TATA box with TTTAAA as the most conserved structure and 3 bp upstream AAA and three bp downstream TTT, although less conserved. TBP is known to bind symmetrically to the TATA box and the orientation of transcription is determined by the binding of TFB (Cox et al., 1997; Bell et al., 1999; Werner and Grohmann, 2011). Therefore, it is possible that some of these antisense transcripts are initiated by opposed TBP binding, which in turn results in two transcripts on opposite strands. An inspection of individual pTSSs using the IGV browser (Robinson et al., 2011) reveals strong signal counts on the antisense strand in short distance upstream of promoter elements for many transcripts. Most striking examples with head-to-tail orientation of the neighboring genes are shown in Figure 5.

**FIGURE 6:**
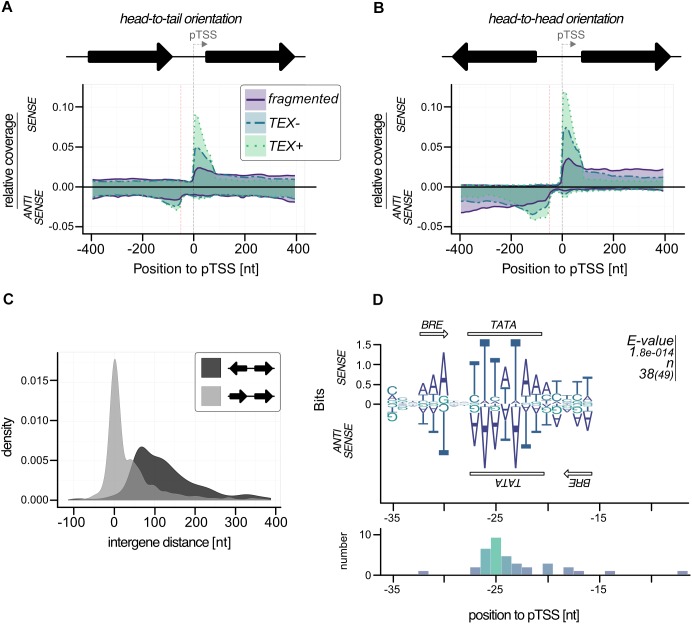
Bidirectional transcription in *P. furiosus.* Genes were sorted in **(A)** head-to-tail or **(B)** head-to-head oriented groups and relative coverage plots (compare Figure 4E) were calculated for both sense and antisense strand. The distance of 50 b upstream of the pTSS is indicated by a red dotted line. **(C)** The intergene distance for both groups is based on gene annotation, head-to-tail orientation are shown in light gray, head-to-head orientation in dark gray. **(D)** MEME motif search for promoter regions with strong antisense signals resulted in a bidirectional BRE-TATA-BRE motif, that is shown on both strands. The *e*-values and the number of sequences contributing to the motif are shown on the right of the panel. This distance to pTSS is shown in the lower panel.

To investigate the occurrence of these bidirectional transcription reads in more detail we plotted the read density for all genes with a detected pTSS from TSSpredator on sense and antisense strand (*n* = 834). To exclude bias from genes in head-to-head orientation with actual pTSS on negative strand we split our dataset according to gene orientation (head-to-head: 388, head-to-tail: 442). This analysis showed a strong antisense peak for head-to-tail orientated genes starting about 50 bp upstream of pTSS (Figure 6A). More than 10% of the 442 genes (49) with this orientation had very strong TEX signals in the region up to 100 bp upstream of the pTSS (more than 40% of the reads from −400 to 400 region). In the case of head-to-head orientated genes, we also observed an enrichment of TEX signals on the antisense strand 50 bp upstream of a pTSS (Figure 6B). The expanded signal distribution is most likely caused by overlapping signals of aTTS and corresponding pTTS of upstream located genes. A more detailed analysis of the intergenic region for head-to-head genes confirmed the short intergene distances (median 117 bp) which impedes any possibility to discriminate between both signals (Figure 6C). The strong accumulation of these antisense transcripts in a distance of approximately 50 bp upstream of the pTSS most likely indicates a shared TATA element for the primary and the corresponding antisense transcript. In this case, we expect an additional BRE element downstream of the TATA element for TFB recruitment in antisense direction. To circumvent the problem with head-to-head orientated genes we only analyzed promoter sequences with head-to-tail orientation. In fact, about 78% of these promoter regions (38) exhibited a bidirectional BRE-TATA-BRE motif located in the middle between a pTSS (position 0) and an aTSS (position −50). The BRE on the antisense strand is less prominent than on the sense strand but can still be detected (Figure 6D).

**FIGURE 7:**
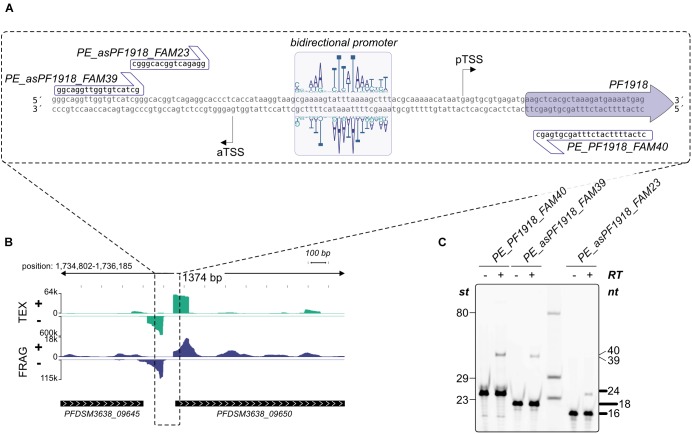
Validation of bidirectional transcripts **(A)** The promoter region of PFDSM3638_09650 (old locus tag PF1918) with the bidirectional motif and the corresponding sequences used for primer extension are specified. **(B)** Zoom out of the promoter region. RNA coverage on both strands is indicated. **(C)** Primer extension analysis of *in vitro* synthesized RNA. The presence (+) or absence (−) of Reverse Transcriptase and the used primers are shown on top of each lane. Lengths of marker fragments (st) are shown on the left, primer signals in bold on the right and expected length of primer extension signals also on the right side.

To get additional evidence for antisense transcription induced by bidirectional promoter sequences, we analyzed the promoter of *PF1918* (PFDSM3638_09650, Figure 5 upper left) in more detail using *in vitro* transcription. A detailed sequence analysis of the upstream region confirmed the presence of a bidirectional promoter (Figure 7A) and the gene upstream of *PF1918* is located in head-to-tail orientation (Figure 7B). To distinguish between sense and antisense transcripts *in vitro*, the RNA was analyzed by primer extension experiments (Figure 7C). A comparison of the 40-nucleotide sense and the 39-nucleotide antisense signal revealed that this bidirectional promoter produces the main transcript as well as the antisense transcript in almost similar amount. The distance between both TSS is 49 bp, which clearly indicates that both transcripts originate from the same TATA element. This is the first *in vitro* evidence in archaea that some of the numerous antisense transcripts can be induced by bidirectional transcription. It is possible that the AT-rich promoter sequence in combination with the low GC content of *P. furiosus* increases the frequency of bidirectional promoters, but we assume that the symmetrical binding of the archaeal TBP to the TATA element (Cox et al., 1997) is especially prone to antisense transcription from bidirectional promoter sequences. This is in line with recent findings in eukaryotes indicating the promoter regions are intrinsically bidirectional and are shaped by evolution to bias transcription toward coding versus non-coding RNAs (Xu et al., 2009; Jin et al., 2017). Furthermore, divergent transcription is a mechanistic feature that does not imply a function for these transcripts. Transcriptional noise as a main result of antisense transcription seems to be also common in bacteria in particular in combination with a high AT content (Lloréns-Rico et al., 2016).

### IS Elements and a Potential Regulation by Antisense Transcripts

**FIGURE 8:**
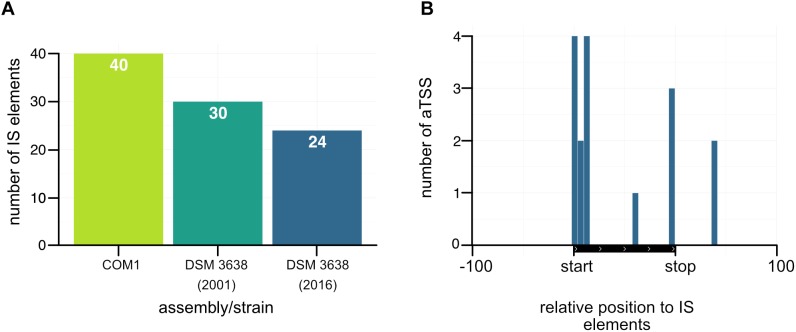
Accumulation of antisense transcripts in the neighborhood of IS elements. **(A)** Comparison of the number of IS elements in the COM1 strain (Bridger et al., 2012) together with the two sequences of the *P. furiosus* type strain. **(B)** Position of aTSSs relative to the transposon coding sequence of IS elements.

As already mentioned in the introduction, *P. furiosus* seems to prone to genomic rearrangements most likely due to an increased number of IS elements, which has been known for a long time as driving force for genomic reorganizations (Sapienza et al., 1982; DiRuggiero et al., 2000; Brügger et al., 2002; Zivanovic et al., 2002). Using ISEScan 1.6 we identified 24 IS elements in the new assembled genome (Figure 8A), whereas the number of IS elements in the “old” strain is 30 and 40 in the COM1 strain. In detail, the number of the IS6 family type of transposable elements is reduced (Table 1). IS6-mediated gene rearrangements have been already described in the early 2002’s as the first genome sequences of different *Pyrococcus* species became available (Zivanovic et al., 2002). Therefore, we assume that the decreased number of IS elements in the new sequenced DSM 3638 strain is a decisive point that ensures genome integrity.

Furthermore, there is increasing evidence that the activity of IS elements could be suppressed by corresponding antisense RNAs in particular under stress conditions (Ellis and Haniford, 2016). As our RNA library pool also contained stress conditions like heat or cold shock and the accumulation of antisense transcripts associated with transposase-encoding genes has also been observed in other archaea (Tang et al., 2002, 2005; Jäger et al., 2009; Straub et al., 2009; Wurtzel et al., 2010; Yoon et al., 2011; Bernick et al., 2012; Heyer et al., 2012; Su et al., 2013; Toffano-Nioche et al., 2013) we mapped aTSS to the relative position of IS elements (Figure 8B). This analysis revealed an increased number of antisense transcripts, which in most cases overlap with the start of the open reading frame of the transposase. Therefore, it seems feasible that antisense transcripts of IS elements might also play a role in gene silencing in *P. furiosus* to avoid genome instability.

## Conclusion

This study provides an updated genome assembly of *P. furiosus* using a combination of long-read PacBio sequencing and short-read Illumina sequencing. The new genome is 18,342 bp smaller than the NCBI reference from 2001 mainly due to a recently described deletion (Reichelt et al., 2016), but the overall structure is still almost identical to the published sequence of *P. furiosus*. The stability of the *P. furiosus* genome was confirmed by re-sequencing of a “lab culture” 2 years after initial sequencing of the strain. Our data demonstrate that it is possible to ensure genome stability in “lab cultures” by avoiding strong selection pressure, even with a strain which was assumed highly susceptible for genome rearrangements (DiRuggiero et al., 2000; Brügger et al., 2002; Zivanovic et al., 2002).

The updated DNA sequence in combination with RNA sequencing enabled us to improve genome annotation using the recently developed pipeline ANNOgesic. We included additional features, such as operon structures, TSSs and terminator sequences as well as non-coding or circRNAs to provide a comprehensive dataset of the genome features of the *P. furiosus* type strain DSM 3638 for future research (Supplementary Figure 2 and Supplementary Table 5).

## Data Availability

Raw sequencing data has been submitted to the NCBI Sequence Read Archive (BioProject: PRJNA382684, BioSample: SAMN06711904). Code, raw figures, and data used during the bioinformatical analysis were uploaded to https://github.com/felixgrunberger/pyrococcus_reannotation.

## Dedication

This paper is dedicated to the memory of our colleague and friend, Prof. Dr. Reinhard Wirth, who recently passed away. Reinhard has identified the LS and the BBR strain of *Pyrococcus furiosus* which exhibit different amounts of flagella and unusual cell morphology.

## Author Contributions

RoR prepared the RNA from *Pyrococcus*. BB, CS, and JO performed the PacBio and Illumina sequencing and FG the nanopore sequencing. The bioinformatical analysis was carried out by FG and RoR. FG, RoR, DG, and WH wrote the manuscript. WH, RR, and DG coordinated and supervised the work. All authors approved the final version of the manuscript.

## Conflict of Interest Statement

The authors declare that the research was conducted in the absence of any commercial or financial relationships that could be construed as a potential conflict of interest.
