# Supplementary figures and images for: Next Generation DNA-Seq and Differential RNA-Seq Allow Re-annotation of the Pyrococcus furiosus DSM 3638 Genome and Provide Insights Into Archaeal Antisense Transcription
Source: Front Microbiol. 2019 Jul 12;10:1603. doi: 10.3389/fmicb.2019.01603 (PMC6640164; doi:10.3389/fmicb.2019.01603)

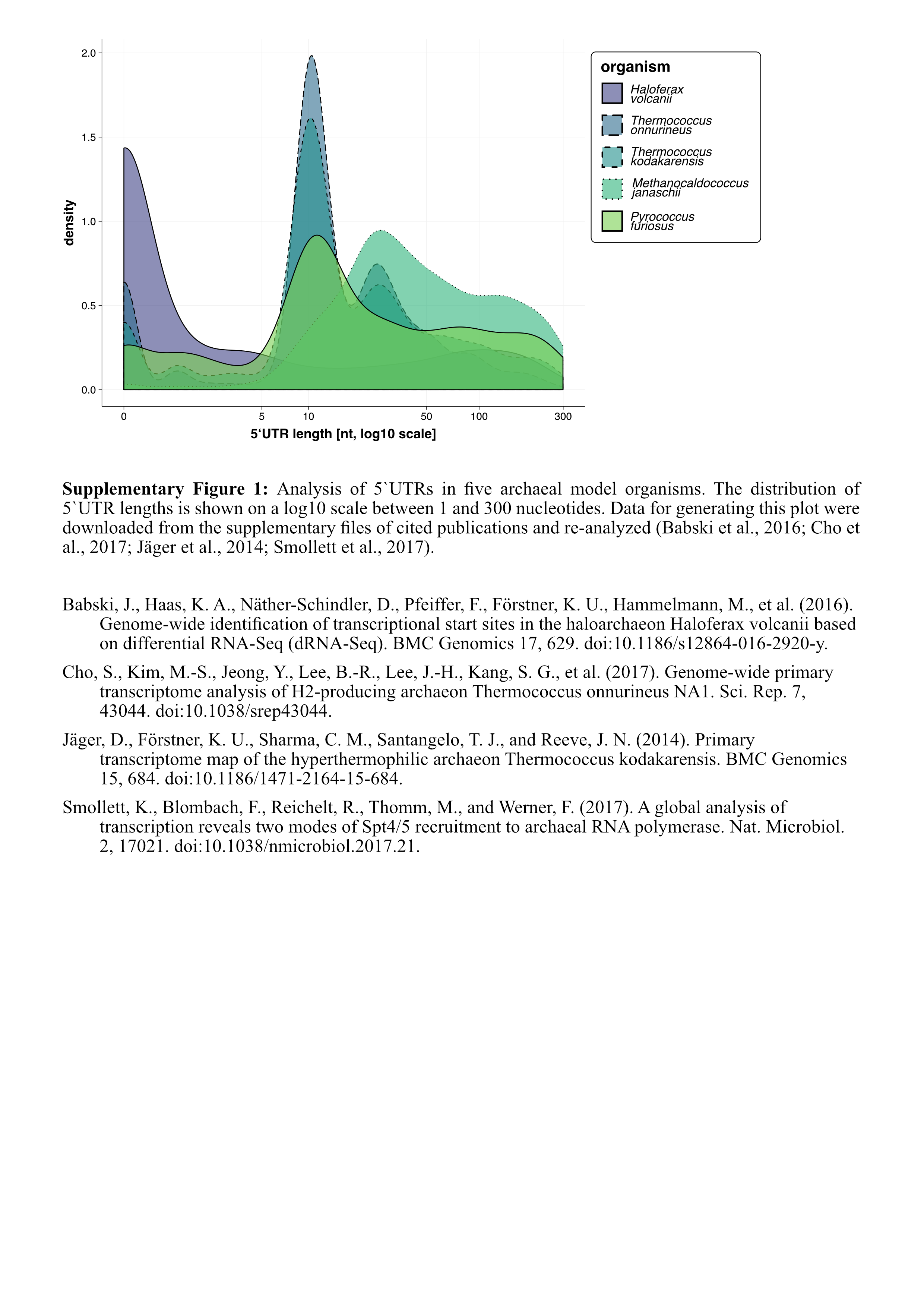

Supplement: Supplementary file 7 [file Image_1.TIFF]

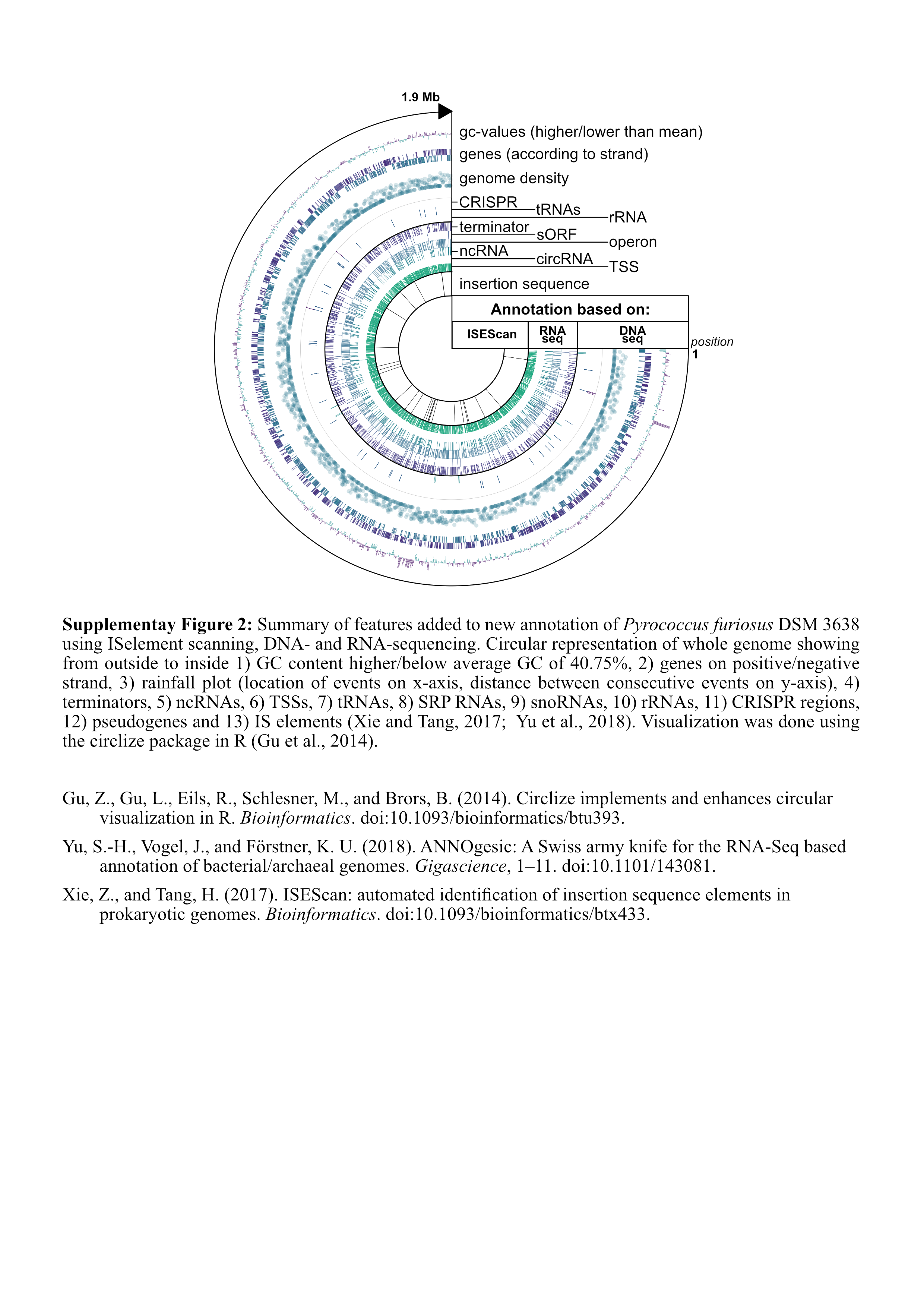

Supplement: Supplementary file 8 [file Image_2.TIFF]
